# Development of a Survival Prognostic Model for Non-small Cell Lung Cancer

**DOI:** 10.3389/fonc.2020.00362

**Published:** 2020-03-20

**Authors:** Yue-Hua Zhang, Yuquan Lu, Hong Lu, Yue-Min Zhou

**Affiliations:** ^1^Department of Oncology, The First Affiliated Hospital of Henan University, Kaifeng, China; ^2^Department of Oncology, Huaihe Hospital of Henan University, Kaifeng, China; ^3^International Joint Research Laboratory for Cell Medical Engineering of Henan, Huaihe Hospital of Henan University, Kaifeng, China

**Keywords:** prognostic model, non-small cell lung cancer, overall survival, hemoglobin, TNM

## Abstract

Lung cancer is a leading cause of cancer-related death, and >80% of lung cancer diagnoses are non-small-cell lung cancer (NSCLC). However, when using current staging and prognostic indices, the prognosis can vary significantly. In the present study, we calculated a prognostic index for predicting overall survival (OS) in NSCLC patients. The data of 545 NSCLC patients were retrospectively reviewed. Univariate and multivariate Cox proportional hazards regression analyses were performed to evaluate the prognostic value of clinicopathological factors. Age (hazard ratio [HR] = 1.25, 95% confidence interval [CI] = 1.02–1.54), TNM stage (III, HR = 1.64, 95% CI = 1.08–2.48; IV, HR = 2.33, 95% CI = 1.48–3.69), lung lobectomy (HR = 1.96, 95% CI = 1.45–2.66), chemotherapy (HR = 1.42, 95% CI = 1.15–1.74), and pretreatment hemoglobin level (HR = 1.61, 95% CI = 1.28–2.02) were independent prognosticators. A prognostic index for NSCLC (PInscl, 0–6 points) was calculated based on age (≥65 years, 1 point), tumor-node-metastasis (TNM) stage (III, 1 point; IV, 2 points), lung lobectomy (no, 1 point), chemotherapy (no, 1 point), and pretreatment hemoglobin level (low, 1 point). In comparison with the “PInscl = 0” subgroup (survival time = 2.71 ± 1.86 years), the “PInscl = 2” subgroup (survival time = 1.86 ± 1.24 years), “PInscl = 3” subgroup (survival time = 1.45 ± 1.07 years), “PInscl = 4” subgroup (survival time = 1.17 ± 1.06 years), “PInscl = 5” subgroup (survival time = 0.81 ± 0.78 years), and “PInscl = 6” subgroup (survival time = 0.65 ± 0.56 years) exhibited significantly shorter survival times. Kaplan-Meier survival analysis showed that patients with higher PInscl scores had poorer OS than those with lower scores (log-rank test: χ^2^ = 155.82, *P* < 0.0001). The area under the curve of PInscl for predicting the 1-year OS was 0.73 (95 % CI = 0.69–0.77, *P* < 0.001), and the PInscl had a better diagnostic performance than the Karnofsky performance status or TNM stage (*P* < 0.01). In conclusion, the PInscl, which is calculated from age, TNM stage, lung lobectomy, chemotherapy, and pretreatment hemoglobin level, significantly predicted OS in NSCLC patients.

## Introduction

Lung cancer is a leading cause of cancer-related death in both men and women (1), and >80% of lung cancer diagnoses are non-small cell lung cancer (NSCLC) (2). To date, the prognosis of NSCLC is mainly based on the tumor-node-metastasis (TNM) staging system (2), histology (2), and predictive biomarker analyses, such as epidermal growth factor (EGFR) mutation (3), anaplastic lymphoma kinase (ALK) translocations (4), c-ros oncogene 1 (*ROS1*) rearrangement (5), and v-raf murine sarcoma viral oncogene homolog B1 ***(****BRAF*) mutation (6). However, the prognosis varies significantly even among patients with the same TNM stage, histomorphological characteristics, and mutation status.

A systematic review (7) of 887 articles and our previous study (8) revealed that there are 169 different clinical and laboratory parameters (including pretreatment hemoglobin and carcinoembryonic antigen levels, performance status, sex, weight, metastases, etc.) and molecular prognostic factors that affect survival in NSCLC patients. However, these clinical and laboratory parameters are inconsistent and not commonly used in clinical practice or trial design. Further, assessing molecular prognostic factors such as EGFR, ALK, *ROS1, BRAF*, and p53 mutation are not only time-consuming but also expensive. Therefore, a practical prognostic model for predicting overall survival (OS) in NSCLC patients is needed. Many prognostic models incorporating various parameters have been reported. These models include the Glasgow prognostic score (GPS) (9), modified GPS (9), laboratory prognostic index (10), and advanced lung cancer inflammation index (11), all of which use serum parameters assessed in routine laboratory tests, but not clinical parameters. Further, Blanchon et al. assessed the prognostic ability of multiple variables, including age, sex, performance status, histological type, and TNM stage, and developed a validated prognostic index (12) in which performance status and TNM stage played major roles.

In the present study, we retrospectively reviewed data from 545 NSCLC patients and calculated a prognostic index (PInscl) for predicting OS in NSCLC patients based on age, TNM stage, lung lobectomy, chemotherapy, and pretreatment hemoglobin levels. The prognostic value of the PInscl was evaluated with receiver operating characteristic (ROC) curve analysis and compared with those of the Karnofsky performance status (KPS) and TNM stage.

## Materials and Methods

### Patients

**Figure 1:**
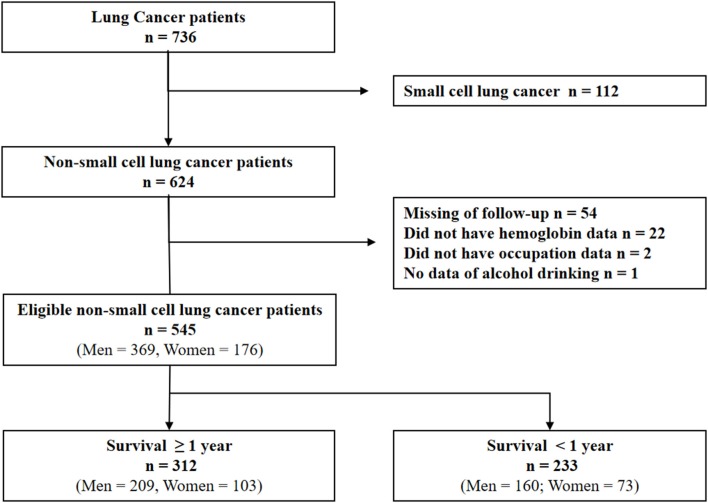
Schematic diagram of participant enrollment in the present study.

All case records of patients with lung cancer admitted to the Huaihe Hospital of Henan University (Henan, China) from May 1, 2010 to June 30, 2017 were analyzed. The inclusion criteria were: (1) NSCLC newly diagnosed at the Huaihe Hospital; (2) histologically or cytologically confirmed NSCLC; and (3) staged according to the TNM staging system (13). Exclusion criteria were: (1) small cell lung cancer; (2) insufficient clinical data; (3) insufficient laboratory data; (4) clinical evidence of active infection or inflammation; (5) hematological disease; (6) pulmonary embolism, acute myocardial infarction, or cerebrovascular accident within 1 month diagnosis. After excluding 191 ineligible patients, 545 patients with NSCLC were selected for the present study (Figure 1). This study was carried out in accordance with the recommendations of the Medical Ethics Committee of Huaihe Hospital, Henan University. The protocol was approved by the Medical Ethics Committee of Huaihe Hospital. All subjects gave written informed consent in accordance with the Declaration of Helsinki.

Data were retrospectively collected from the patients' case records, including demographic information (age, sex, cigarette smoking, alcohol consumption, and family history of cancer), date of diagnosis and death (obtained from the patients' medical records, local death registration departments, and telephone follow-ups), cancer stage at the time of diagnosis (according to the 8th Edition of the TNM Classification for Lung Cancer) (13), KPS score (≥80 indicated that the patient was able to live and work with mild symptoms or signs and <80 indicated that the patient was unable to live and work normally) (14), therapeutic method (obtained from the patients' medical records), and pretreatment hemoglobin levels [<120 g/L was defined as low pretreatment hemoglobin (LPHb) in men and <110 g/L was defined as LPHb in women according to the normal reference range of hemoglobin in the Chinese population].

### Follow-Up

Patients with NSCLC were followed from the date of diagnosis to the date of death or June 25, 2017, whichever came first. OS for each patient was defined as the number of days from the date of diagnosis to the date of death or final follow-up. Person-years were calculated for each subject. Treatments were initiated upon diagnosis and the treatment methods were not exclusive; a patient may have undergone lobectomy, chemotherapy, and radiation simultaneously.

### Statistical Analysis

For univariate and multivariate Cox proportional hazards regression analysis, age (<65 vs. ≥65), sex, TNM stage (I-II vs. III-IV), KPS score (≥80 vs. <80), lung lobectomy status, chemotherapy, radiotherapy, smoking status, alcohol consumption, family history of cancer, and pretreatment hemoglobin levels (normal pretreatment hemoglobin (NPHb) vs. LPHb were categorized into the reference group and the observed group, with hazard ratios (HR) and 95% confidence intervals (CI) being calculated to estimate associations between the observed factors and OS in patients with NSCLC. After discarding the insignificant factors in the multivariate analysis, the final Cox model included age, TNM stage, lung lobectomy, chemotherapy, and pretreatment hemoglobin. Between two prognostic factors, an interaction effect was tested using multivariate analysis. For each enrolled item, proportionality was estimated using the Schoenfeld and scaled Schoenfeld residuals.

We developed a PInscl that included age, TNM stage, lung lobectomy, chemotherapy, and pretreatment hemoglobin based on the results of the final Cox model. Age ≥ 65 years, TNM stage III, not undergoing lung lobectomy, not receiving chemotherapy, and having LPHb were given 1 point; TNM stage IV was given 2 points. The minimum PInscl score was 0 and the maximum PInscl score was 6 (Supplementary Table 1). The OS, HR, and 95% CI were calculated for each PInscl score. Associations between PInscl score and OS were evaluated using the Peto-Peto-Prentice test. Survival curves were generated using the Kaplan-Meier method, and the log-rank test was used to examine differences in OS between patients with different PInscl scores.

The discriminatory ability of the PInscl score was tested by assessing the area under the ROC curve (AUC). Further, the AUC of PInscl was compared with those of the KPS and TNM staging using the *DeLong* test (15). In addition, we calculated the sensitivity, specificity, negative predictive value (NPV), and positive predictive value (PPV) of the prognostic score.

All statistical analyses were performed using Stata software version 13 (Stata Corporation, College Station, TX, USA). *P* < 0.05 was considered to indicate a statistically significant difference for all analyses.

## Results

### Patient Characteristics

Our study included a total of 545 NSCLC patients including 369 men and 176 women. Over half (53.2%) of the patients were <65 years, 59.4% had a KPS score ≥80, 41.7% had stage III disease, and 44.4% had stage IV disease. Approximately a quarter (22.4%) of the patients had LPHb. Treatment methods included lung lobectomy (*n* = 164, 30.1%), chemotherapy (*n* = 282, 51.7%), and radiotherapy (*n* = 90, 16.5%) (Table 1).

**Table 1:** Clinicopathological and lifestyle factors for patients with non-small cell lung cancer.

		**No. of subjects (%)**		
		**Overall survival <1 year**	**Overall survival ≥ 1 year**	***P*-value^**a**^**
Age (years), median ± SD		63.9 ± 10.7	62.0 ± 9.3	0.036
	<65	113 (48.5)	177 (56.7)	0.057
	≥65	120 (51.5)	135 (43.3)	
Sex				
	Male	160 (68.7)	209 (67.0)	0.678
	Female	73 (31.3)	103 (33.0)	
TNM stage				
	I-II	11 (4.7)	65 (20.8)	<0.001
	III	84 (36.1)	143 (45.8)	
	IV	138 (59.2)	104 (33.3)	
KPS score				
	≥80	112 (48.1)	212 (68.0)	<0.001
	<80	121 (51.9)	100 (32.1)	
Lung lobectomy				
	Yes	29 (12.5)	135 (43.3)	<0.001
	No	204 (87.6)	177 (56.7)	
Chemotherapy				
	Yes	94 (40.3)	188 (60.3)	<0.001
	No	139 (59.7)	124 (39.7)	
Radiotherapy				
	Yes	29 (12.5)	61 (19.6)	0.027
	No	204 (87.6)	251 (80.5)	
Cigarette smoking				
	No	102 (43.8)	136 (43.6)	0.965
	Yes	131 (56.2)	176 (56.4)	
Alcohol consumption				
	No	186 (79.8)	253 (81.1)	0.713
	Yes	47 (20.2)	59 (18.9)	
Family history				
	No	217 (93.1)	291 (93.3)	0.950
	Yes	16 (6.9)	21 (6.7)	
Hemoglobin, g/L, median ± SD		122.9 ± 20.3	130.3 ± 14.4	<0.001
	NPHb	158 (67.8)	265 (84.9)	<0.001
	LPHb	75 (32.2)	47 (15.1)	

*Data are presented as n (%) unless otherwise noted*.

a *Chi square test. SD, standard deviation; TNM, tumor-node-metastasis; KPS, Karnofsky performance status; NPHb, normal pretreatment hemoglobin (men, 120–160 g/L; women, 110–150 g/L); LPHb, low pretreatment hemoglobin (men, <120 g/L; women, ≤110 g/L)*.

### Univariate Analysis

On univariate Cox proportional hazards regression analysis, a significantly longer survival was observed in patients aged <65 years at diagnosis (HR = 1.42, 95% CI = 1.18–1.73) and who had stage I-II disease (compared to patients with stage III disease, HR = 2.62, 95% CI = 1.81–3.79 or stage IV disease, HR = 4.39, 95% CI = 3.04–6.33). Further, a KPS score ≥80 (HR = 1.85, 95% CI = 1.52–2.25), lung lobectomy (HR = 3.10, 95% CI = 2.44–3.94), chemotherapy (HR = 1.63, 95% CI = 1.34–1.98), radiotherapy (HR = 1.31, 95% CI=1.01–1.70), and NPHb (HR = 1.82, 95% CI = 1.45–2.27) significantly improved prognosis. However, there was no significant association between OS and sex (HR = 1.00, 95% CI = 0.82–1.23), cigarette smoking (HR = 0.98, 95% CI = 0.81–1.20), alcohol consumption (HR = 1.04, 95% CI = 0.82–1.32), or a family history of cancer (HR = 0.91, 95% CI = 0.61–1.36) (Table 2).

**Table 2:** Univariate and multivariate analysis of prognostic factors for patients with non-small cell lung cancer.

**Prognostic factor**		**No. of subjects**	**Univariate**^****a****^			**Multivariate**^****b****^		
			**HR**	**95% CI**	***P*-value**	**HR**	**95% CI**	***P*-value**
**Total**								
Age	<65	290	1.00			1.00		
	≥65	255	1.42	1.18–1.73	<0.001	1.23	1.00–1.52	0.052
Sex	Male	369	1.00			1.00		
	Female	176	1.00	0.82–1.23	0.972	1.09	0.79–1.50	0.608
TNM Stage	I-II	76	1.00			1.00		
	III	227	2.62	1.81–3.79		1.62	1.06–2.45	0.024
	IV	242	4.39	3.04–6.33	<0.001	2.31	1.45–3.68	<0.001
KPS score	≥80	324	1.00			1.00		
	<80	221	1.85	1.52–2.25	<0.001	1.14	0.92–1.40	0.239
Lung lobectomy	Yes	164	1.00			1.00		
	No	381	3.10	2.44–3.94	<0.001	1.93	1.42–2.63	<0.001
Chemotherapy	Yes	282	1.00			1.00		
	No	263	1.63	1.34–1.98	<0.001	1.41	1.13–1.76	0.002
Radiotherapy	Yes	90	1.00			1.00		
	No	455	1.31	1.01–1.70	0.044	1.04	0.78–1.38	0.811
Smoking	No	238	1.00			1.00		
	Yes	307	0.98	0.81–1.20	0.870	1.20	0.88–1.63	0.239
Alcohol consumption	No	439	1.00			1.00		
	Yes	106	1.04	0.82–1.32	0.757	1.07	0.82–1.38	0.622
Family history	No	508	1.00			1.00		
	Yes	37	0.91	0.61–1.36	0.638	0.90	0.60–1.36	0.630
Hemoglobin	NPHb	432	1.00			1.00		
	LPHb	122	1.82	1.45–2.27	<0.001	1.54	1.22–1.94	<0.001

a *For univariate analysis, Cox proportional-hazards model included survival time (<1 or ≥1 year) and one of the following factors: Age, sex, TNM stage, KPS score, lung lobectomy, chemotherapy, radiotherapy, smoking, alcohol consumption, family history or hemoglobin*.

b *For multivariate analysis, Cox proportional-hazards model included survival time (<1 or ≥1 year), age, sex, TNM stage, KPS score, lung lobectomy, chemotherapy, radiotherapy, smoking, alcohol consumption, family history, and hemoglobin. HR, hazard ratio; CI, confidence interval; TNM, tumor-node-metastasis; KPS, Karnofsky performance status; NPHb, normal pretreatment hemoglobin (men, 120–160 g/L; women 110–150 g/L); LPHb, low pretreatment hemoglobin (men, <120 g/L; women, ≤110 g/L)*.

### Multivariate Analysis

Multivariate Cox proportional hazards regression analysis showed that age ≥65 (HR = 1.23, 95% CI = 1.00–1.52), TNM stage (III, HR = 1.62, 95% CI = 1.06–2.45; IV, HR = 2.31, 95% CI = 1.45–3.68), lung lobectomy (HR = 1.93, 95% CI = 1.42–2.63), chemotherapy (HR = 1.41, 95% CI = 1.13–1.76), and LPHb (HR = 1.54, 95% CI = 1.22–1.94) were independently significantly associated with decreased OS (Table 2).

The final Cox model indicated that age ≥65 (HR = 1.25, 95% CI = 1.02–1.54), TNM stage (III, HR = 1.64, 95% CI = 1.08–2.48; IV, HR = 2.33, 95% CI = 1.48–3.69), lung lobectomy (HR = 1.96, 95% CI = 1.45–2.66), chemotherapy (HR = 1.42, 95% CI = 1.15–1.74), and LPHb (HR = 1.61, 95% CI = 1.28–2.02) were significantly independent unfavorable prognostic factors of 1-year survival in patients with NSCLC (Table 3).

**Table 3:** Prognostic factors included in the final Cox proportional hazard model for prediction of 1-year survival of 545 patients with non-small cell lung cancer.

**Prognostic factor**		**No. of subjects**	**HR**	**95% CI**	***P*-value**
Age	<65	290	1.00		
	≥65	255	1.25	1.02–1.54	0.030
TNM Stage	I–II	76	1.00		
	III	227	1.64	1.08–2.48	0.020
	IV	242	2.33	1.48–3.69	<0.001
Lung lobectomy	Yes	164	1.00		
	No	381	1.96	1.45–2.66	<0.001
Chemotherapy	Yes	282	1.00		
	No	263	1.42	1.15–1.74	0.001
Hemoglobin	NPHb	423	1.00		
	LPHb	122	1.61	1.28–2.02	<0.001

*HR, hazard ratio by multivariate Cox proportional hazards regression; CI, confidence interval; TNM, tumor-node-metastasis; NPHb, normal pretreatment hemoglobin (men, 120–160 g/L; women, 110–150 g/L); LPHb, low pretreatment hemoglobin (men, <120 g/L; women, ≤110 g/L)*.

### Prognostic Index for Non-small Cell Lung Cancer (PInscl)

**Figure 2:**
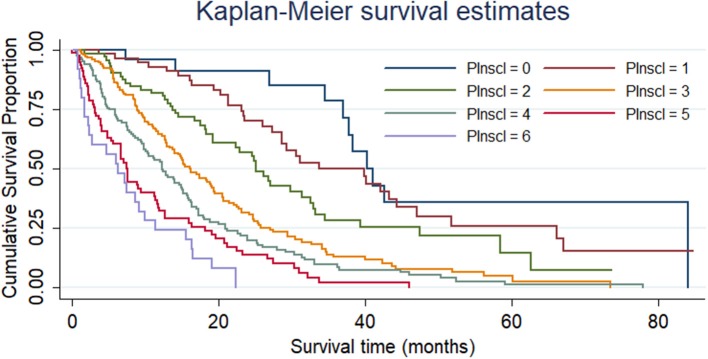
Cumulative survival of patients with non-small cell lung cancer according to the PInscl. Patients with higher PInscl scores (refer to Supplementary Table) exhibited a poorer overall survival than those with lower PInscl scores (log-rank test, χ^2^ = 155.82; *P* < 0.0001).

In comparison with the “PInscl = 0” subgroup (survival time = 2.71 ± 1.86 years), the “PInscl = 2” subgroup (survival time = 1.86 ± 1.24 years; HR = 2.36, 95% CI = 1.21–4.59), “PInscl = 3” subgroup (survival time = 1.45 ± 1.07 years; HR = 4.18, 95% CI = 2.23–7.82), “PInscl = 4” subgroup (survival time = 1.17 ± 1.06 years; HR = 5.69, 95% CI = 3.03–10.66), “PInscl = 5” subgroup (survival time = 0.81 ± 0.78 years; HR = 8.75, 95% CI = 4.57–16.76), and “PInscl = 6” subgroup (survival time = 0.65 ± 0.56 years; HR = 13.13, 95% CI = 6.32–27.27) had a significantly shorter survival time (Table 4). Kaplan-Meier survival curve analysis showed that patients with higher PInscl scores had a poorer OS than those with lower scores (log-rank test, χ^2^ = 155.82; *P* < 0.0001) (Figure 2).

**Table 4:** Combined prognostic effects of age, TNM stage, lung lobectomy, chemotherapy, and pretreatment hemoglobin levels for 545 patients with non-small cell lung cancer.

**PInscl^**a**^**	**No. of subjects**	**Survival time, years** **(Mean ± SD)**	**HR**	**95% CI**	***P*-value**
Total	545	1.47 ± 1.27			
0	26	2.71 ± 1.86	1.00		
1	59	2.43 ± 1.53	1.48	0.75–2.95	0.261
2	73	1.86 ± 1.24	2.36	1.21–4.59	0.012
3	151	1.45 ± 1.07	4.18	2.23–7.82	<0.001
4	131	1.17 ± 1.06	5.69	3.03–10.66	<0.001
5	80	0.81 ± 0.78	8.75	4.57–16.76	<0.001
6	25	0.65 ± 0.56	13.13	6.32–27.28	<0.001

a *PInscl, prognostic index for non-small cell lung cancer (ref. Supplementary Table 1), P-value for trend, <0.0001 (Peto-Peto-Prentice test). SD, standard deviation; HR, hazard ratio by multivariate Cox proportional hazards regression; CI, confidence interval; NPHb, normal pretreatment hemoglobin (men, 120–160 g/L; women, 110–150 g/L); LPHb, low pretreatment hemoglobin (men, <120 g/L; women, ≤110 g/L)*.

**Figure 3:**
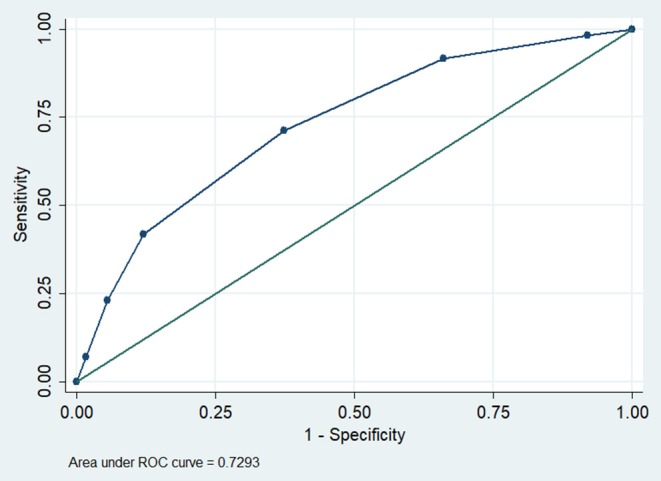
Discriminatory power for PInscl predicting 1-year overall survival (OS). The area under the curve (AUC) was 0.73 (95 % confidence interval = 0.69–0.77, *P* < 0.0001).

The AUC for the PInscl for predicting 1-year OS was 0.73 (95% CI = 0.69–0.77, *P* < 0.001) (Figure 3). Comparisons of the AUCs between the PInscl and the KPS or the TNM stage showed that the PInscl had a better diagnostic performance than either the KPS or the TNM stage (Table 5). The sensitivity, specificity, NPV, and PPV for the PInscl index were 71.2, 62.7, 71.8, and 61.9%, respectively.

**Table 5:** Discriminatory power of the PInscl, KPS, and TNM for overall survival of non-small cell lung cancer patients.

**Area under the curve (AUC)**					
**Overall survival**	**1-year**	**2-year**	**3-year**	**4-year**	**5-year**
PInscl	0.73 ± 0.02 (0.69–0.77)	0.73 ± 0.02 (0.68–0.77)	0.77 ± 0.03 (0.71–0.83)	0.75 ± 0.04 (0.66–0.83)	0.83 ± 0.06 (0.72–0.94)
KPS	0.64 ± 0.02 (0.59–0.68)^**^	0.59 ± 0.03 (0.54–0.64)^**^	0.63 ± 0.03 (0.57–0.7)^**^	0.600± 0.05 (0.49–0.7)^**^	0.76 ± 0.06 (0.64–0.87)^**^
TNM	0.67 ± 0.02 (0.63–0.71)^**^	0.67 ± 0.03 (0.62–0.73)^**^	0.70 ± 0.03 (0.64–0.77)^**^	0.66 ± 0.05 (0.55–0.76)^*^	0.69 ± 0.08 (0.54–0.85)^**^

*PInscl, prognostic index for non-small cell lung cancer; KPS, Karnofsky performance status; TNM, tumor-node-metastasis stage*.

* *p < 0.05*,

** *p < 0.01*.

## Discussion

The results of the present study highlighted the importance of prognostic models in estimating prognosis in NSCLC patients. Our prognostic model, the PInscl, was based on age, TNM stage, lung lobectomy, chemotherapy, and pretreatment hemoglobin level. The PInscl had a statistically significant discriminative ability to predict OS. Further, the PInscl had a statistically better diagnostic performance than the KPS score or the TNM stage for 1–5 year OS (Table 5). This might be because the PInscl included other factors, making it more comprehensive and sensitive.

In previous studies, age has been recognized as a prognostic factor for NSCLC using cut-off values of 80, 75, 70, and even 50 years (16–19). In the present study, age <65 years was associated with a longer survival time in both univariate (HR = 1.42, 95% CI = 1.18–1.73) and multivariate (HR = 1.23, 95% CI = 1.00–1.52) analyses. We also analyzed age as a continuous variable, but it was not significantly correlated with OS.

The TNM staging system, which classifies cancer according to the size and extension of the primary tumor, its lymphatic involvement, and the presence of metastases, is frequently used in clinical practice to predict prognosis (20). Its reliability has been fully established through the IASLC (International Association for the Study of Lung Cancer) study (21). In our present study, stage III (HR = 1.64, 95% CI = 1.08–2.48) and stage IV (HR = 2.33, 95% CI = 1.48–3.69) disease were indicative of a poorer prognosis (Table 3). However, as the coefficient of the TNM stage was not more than two times those of other factors in multivariate analysis (data not shown), we did not emphasize it in our model, as Blancoon et al. did (12).

Anemia is linked to prognosis, and hemoglobin has long been recognized as a prognostic factor for NSCLC patients (22–25). We found that hemoglobin <120 g/L in men and <110 g/L in women was associated with a shorter OS (HR = 1.62, 95% CI = 1.29–2.03).

In many cases, lung lobectomy is still the most effective treatment method for NSCLC (26). The impact of minimally invasive lobectomy and thoracotomy lobectomy on survival has also been assessed (27). However, lobectomy will be applied according to the clinical situation for NSCLC patients (28). In the present study, surgical resection was not recommended for stage IV patients. Therefore, although we found that lung lobectomy was an independent prognostic factor for NSCLC patients, we cannot say whether a physical condition suitable for lobectomy, lobectomy itself, or both contributed favorably to OS. Regardless, lung lobectomy was an independent prognostic factor in the model.

Chemotherapy is another major treatment method for NSCLC (29), and more chemotherapies have become clinically available (30). We found that chemotherapy was an independent prognostic factor in both univariate and multivariate analysis. This result was in line with those of previous studies (8, 31) However, patients received both cisplatin- and paclitaxel-based chemotherapies, and we did not divide the patients into subgroups, which may have affected the results. Chemotherapy, particularly cisplatin-based adjuvant chemotherapy, might also improve survival among patients with completely resected NSCLC (32). Although we could not exclude its potential long-term influence, we did not find a significant synergistic effect of chemotherapy and lung lobectomy (data not shown).

This study has several strengths. First, the PInscl can be simply calculated and used in almost all NSCLC patients. Data on age, TNM stage, lung lobectomy, chemotherapy, and pretreatment hemoglobin are easy to obtain and do not require exhaustive testing and complicated biological examination. Second, it is practicable. We could predict OS simply by the PInscl score, which is meaningful for patients, their families, and clinicians. ROC curve analysis showed that the PInscl score was a fairly predictable index and was more sensitive than the KPS and TNM score. However, the study also has limitations. First, selection bias may be a concern due to the monocentric design of the study and the absence of random sampling, even though exhaustive inclusion of consecutive cases over 5-years should alleviate the bias. Second, the discriminative power of the PInscl was not assessed in a population with features different from that in which it was derived. Third, the model does not include mutational information (e.g., EGFR/ALK mutations). Fourth, the lack of a validation cohort might weaken the power of the present study. Therefore, whether it is suitable to be expostulated to other NSCLC populations needs further verification.

By developing this simple prognostic index, we suggest that the PInscl, which is calculated from age, TNM stage, lung lobectomy, chemotherapy, and pretreatment hemoglobin level, might significantly predict OS in NSCLC patients.

## Data Availability Statement

The datasets analyzed in this article are not publicly available. Requests to access the datasets should be directed to Yuquan Lu ( lll3923@gmail.com).

## Ethics Statement

The studies involving human participants were reviewed and approved by the Medical Ethics Committee of Henan University, Huaihe Hospital. Written informed consent for participation was not required for this study in accordance with the national legislation and the institutional requirements.

## Author Contributions

Y-HZ made substantial contributions to data collection and was a major contributor in writing the manuscript. YL analyzed and interpreted the data contributed to manuscript preparation and revision and gave final approval for the version to be published. HL was responsible for the acquisition of data and institutional review board application, conducted data interpretation, and gave final approval for the version to be published. Y-MZ agreed to be accountable for all aspects of the work in ensuring that questions related to the accuracy or integrity of any part of the work are appropriately investigated and resolved.

### Conflict of Interest

The authors declare that the research was conducted in the absence of any commercial or financial relationships that could be construed as a potential conflict of interest.

## Abbreviations

NSCLC: non-small cell lung cancer
TNM: tumor-node-metastasis
EGFR: epidermal growth factor receptor
ALK: anaplastic lymphoma kinase
ROS1: c-ros oncogene 1
BRAF: v-raf murine sarcoma viral oncogene homolog B1
OS: overall survival
GPS: Glasgow prognostic score
ROC: receiver operative characteristic
KPS: Karnofsky performance status
LPHb: low pretreatment hemoglobin
NPHb: high pretreatment hemoglobin
HR: hazard ratio
CI: confidence interval
AUC: area under the curve
NPV: negative predictive value
PPV: positive predictive value
IASLC: International Association for the Study of Lung Cancer.
